# Potential Role of JAK Inhibitors in the Treatment of Systemic Sclerosis-Associated Interstitial Lung Disease: A Narrative Review from Pathogenesis to Real-Life Data

**DOI:** 10.3390/life12122101

**Published:** 2022-12-14

**Authors:** Elisa Fiorentini, Francesco Bonomi, Silvia Peretti, Martina Orlandi, Gemma Lepri, Marco Matucci Cerinic, Silvia Bellando Randone, Serena Guiducci

**Affiliations:** 1 Department of Clinical and Experimental Medicine, University of Florence, 50134 Florence, Italy; 2 Unit of Immunology, Rheumatology, Allergy and Rare Diseases (UnIRAR), IRCCS San Raffaele Hospital, 20132 Milan, Italy

**Keywords:** systemic sclerosis, interstitial lung disease, JAK inhibitors, autoimmune disease

## Abstract

Background: Systemic sclerosis-associated interstitial lung disease (SSc-ILD) is one of the most relevant complications of SSc and the major cause of death. The pathogenesis of SSc-ILD involves a complex interplay of multiple cell types and different molecular pathways, with both inflammation and fibrosis as pathological hallmarks. To date, there are no treatments able to target both components of the disease. Janus kinase inhibitors (JAKinibs) represent an interesting therapeutic option because they exert both anti-inflammatory and anti-fibrotic properties. Methods: Here, we performed a narrative review concerning the potential role of JAKinibs in SSc-ILD to define the state of art and to evaluate the pathogenetic rationale behind this type of treatment. Results: Currently, few studies investigated SSc-ILD response to JAKinibs treatment. Data were analyzed from three clinical studies and four case reports and progression of SSc-ILD was not evident in 93.5% of patients treated with JAKinibs. Conclusions: Available evidence of efficacy of JAKinibs in SSc-ILD is sparse but promising. JAKinibs could be an interesting treatment in SSc-ILD because of their potential inhibition of the fibrotic processes combined with their anti-inflammatory action. Moreover, JAKinibs were also shown in some studies to have a potential effect on pulmonary arterial hypertension (PAH), another threatening complication in SSc. More data are necessary to define JAKinibs role in SSc-ILD treatment.

## 1. Introduction

Systemic sclerosis (SSc) is a rare, chronic, and heterogeneous connective tissue disease, characterized by the association of vascular damage, dysregulation of the immune system, and fibrosis [1]. Interstitial lung disease (SSc-ILD) is one of the most severe complications and, to date, the major cause of death [2]. According to the histopathological pattern, ILD is mainly classified into non-specific interstitial pneumonia (NSIP) and usual interstitial pneumonia (UIP). In SSc, NSIP is the most frequent pattern (around 76% of cases) and is characterized by homogeneous lesions with predominant inflammatory infiltrates without much destruction or fibrosis. Instead, UIP is seen in about 11% of SSc-ILD and corresponds to heterogeneous lesions with a lesser degree of inflammatory infiltrates, and foci of young fibroblastic tissue at different stages of evolution [3,4]. Treatment options with disease-modifying drugs are limited, and this may be explained partly by the incomplete knowledge of the mechanisms underlying lung fibrosis development and the heterogeneous course of the disease.

The pathogenesis of SSc-ILD involves a complex interplay between multiple cell types and several molecular pathways, with both inflammation and fibrosis as pathologic hallmarks. One of the possible pathogenetic models starts with an injury—either to the lung epithelium or to the endothelium of the pulmonary vessels—which, in patients with a genetic predisposition and often with immune dysregulation, leads to infiltration of inflammatory cells into the alveolar membrane and airspaces [5]. Under these stimuli, lung-resident immune cells are activated and proliferate, and other inflammatory cells (monocytes, neutrophils, mast cells, and natural killer (NK) cells) are recruited and infiltrate lung tissues. Levels of pro-inflammatory cytokines and chemokines (such as interleukins (IL-8, IL-1α, IL-10), macrophage inflammatory protein-1α (MIP-1α), monocyte chemoattractant protein-1 (MCP-1), and neutrophil-derived alpha-defensin) have been proven to be increased in BALF of SSc-ILD patients [6], as have other pro-inflammatory mediators (such as tumor necrosis factor-α (TNFα), IL-6 and interferon-γ (IFNγ)) are increased in plasma of SSc-ILD patients [7,8].

Interstitial pulmonary fibroblasts are stimulated in this particular environment characterized by inflammation and epithelial damage, and they overproduce many signaling peptides involved in the fibrotic process, promoting consequent fibrosis affecting endothelial cells and lung vessels, leading to airspaces and peripheral airways damage [9]. Transforming growth factor β (TGF-β) is the central regulator of this process, increasing the expression of profibrotic and pro-inflammatory cytokines and growth factors (such as TNF-α, platelet-derived growth factor (PDGF), IL-1β, and IL-13), promoting a profibrotic microenvironment in lungs and stimulating epithelial-to-mesenchymal transition (EMT) and endothelial-to-mesenchymal transition (endoMT) [2]. Chemokine ligand 18 (CCL18) is another interesting molecule in the fibrotic process of SSc-ILD; while its role is not yet fully understood, it seems to drive fibroblast proliferation and collagen production, and its levels are increased in BALF of SSc-ILD patients [6,7]. This profibrotic environment causes aberrant alveolar repair responses with the creation of fibroblast foci and excessive production of extracellular matrix (ECM), leading to extensive remodeling of lung parenchyma and subsequent damage of its function.

Nowadays, there are many experimental animal models regarding human ILD, but none of them entirely illustrate human disease from onset to progression. In the future, new helpful insights might be brought by human preclinical models, such as organoids, possibly leading to the understanding of pathogenic mechanisms and the discovery of new targets for personalized treatment of SSc-ILD [2].

Knowing that both inflammation and fibrosis have a pivotal role in this complex pathogenetic process, the recent pharmacological class of Janus Kinase Inhibitors (also known as JAKinibs [10]) may represent an interesting option for the treatment of SSc-ILD. Janus kinases (JAK) are four intracellular tyrosine kinases (JAK1, JAK2, JAK3, TYK2), broadly expressed in many tissues and cells, that are fundamental in the signaling cascade that follows the activation of many different membrane receptors. Signal Transducers and Activators of Transcription (STATs) are instead a group of seven members (STAT1, STAT2, STAT3, STAT4, STAT5a, STAT5b, STAT6), with the role of transcription factors [11]. JAKinibs interfere with JAK/STAT pathway undergoing competitive ATP binding, impeding the phosphorylation of cytokine receptors and blocking gene transcription; this results in reduced production of multiple cytokines and compromised differentiation of immune cells [12]. Nowadays, there are several JAK inhibitors used for the treatment of various autoimmune and malignant diseases. To date, they are divided in two generations, the first being made of pan-JAKinibs (tofacitinib, baricitinib, ruxolitinib, peficitinib) and the second of selective JAKinibs (decernotinib–JAK3, filgotinib–JAK1, upadacitinib–JAK1) [13].

JAKinibs exert proven anti-inflammatory properties in rheumatoid arthritis (RA) thanks to their effects on the plasma levels of pro-inflammatory cytokines as IL-6 and IL-17 [14,15]. In addition, JAKinibs can modulate the pro-inflammatory state of M1 macrophages diminishing interferon β (IFNβ) signature and IL-6 production [16]. Beyond these anti-inflammatory effects, JAKinibs also exert anti-fibrotic properties in other fibrotic disorders (i.e., autoimmune liver fibrosis or idiopathic pulmonary fibrosis), possibly due to their direct impact on fibroblast activation, interfering with the production of many profibrotic cytokines (as IL-12, IL-4, IL-5) [17,18,19]. However, the precise impact of JAKinibs on the complex spectrum of activation of macrophages is still to be defined, particularly in disorders where both pro-inflammatory M1 macrophages and profibrotic M2 macrophages are excessively activated (such as in SSc-ILD) [20].

This evidence prompted us to investigate the use of JAKinibs in SSc-ILD.

## 2. Materials and Methods

We performed a narrative (PRISMA protocol not followed) non-systematic review of the state of art on PubMed using the keywords: “systemic sclerosis”, “scleroderma”, “jak inhibitors”, “Janus kinase inhibitors”, “tofacitinib”, “baricitinib”, “upadacitinib”, “filgotinib” and “interstitial lung disease”, “lung fibrosis”, “pulmonary fibrosis”. No time limit was put on the research. Only JAKinibs that are routinely used in rheumatological practice were investigated. Clinical trials and case reports were included while literature reviews and animal studies were excluded. We also checked the reference list of each paper for additional studies.

## 3. Results

Our literature search retrieved seven articles that were published between 2018 and 2022. Of these, You et al. [21] conducted a single-center, open-label clinical trial, Karalilova et al. [22] conducted a pilot study, Hou et al. [23] conducted a prospective observational study, and the remaining were case reports [24,25,26,27]. The extracted data were sparse and not complete because treatment with JAKinibs was mainly started for a cutaneous or an articular involvement. The studies used only tofacitinib or baricitinib. No publication investigating for SSc the use of filgotinib or upadacitinib was found. Table 1 reports a summary of the analyzed articles.

### 3.1. Patients Characteristics

A general overview was possible only on patients from six studies (19/32), since Karalilova et al. did not perform a subanalysis of the characteristics of the 13 patients with SSc-ILD.

- Deverapalli et al. discussed a case of a 27-year-old male with SSc and mild restrictive lung disease at pulmonary function tests: he was previously treated with MMF and received treatment with tofacitinib [24];
- Fujita et al. presented a 71-year-old woman with SSc, RA, and type 1 diabetes, previously treated with MTX, TCZ, and adalimumab (ADA). She had rheumatoid factor (RF) and anti-citrullinated peptide (anti-CCP) autoantibodies positivity and mild fibrosis involving the lower lobes at HRCT. The patient was treated with baricitinib [25];
- You et al. analyzed tofacitinib response in 10 SSc patients (5 females and 1 man with ILD at high-resolution CT (HRCT) (mean age 57 years). Autoantibodies were not available and 2/6 did not receive previous immunosuppressive treatment, while the other 4 were previously treated with corticosteroids (CS, 3/4), methotrexate (MTX, 1/4), mycophenolate mofetil (MMF, 3/4), and cyclophosphamide (CYC, 1/4) [21];
- Boleto et al. presented a 48-year-old man with overlap SSc (anti-Scl70 positive) and RA with RF and anti-CCP positivity. He had minimal ILD at HRCT (no definition of minimal was given but it usually refers to an ILD involving <5% of the total lung volume [28]). No clear differentiation between SSc-ILD and RA-ILD was provided. The patient was previously treated with CS, MTX, rituximab (RTX), tocilizumab (TCZ), and abatacept (ABA). The patient was treated with baricitinib [26];
- Kyriakou et al. reported the case of a 58-year-old woman with SSc and axial spondyloarthritis with anti-Scl70 positivity. The patient showed mild fibrosis of the lower lobes at HRCT and was previously treated with corticosteroids, MMF, TCZ and etanercept. The patient was then treated with tofacitinib [27];
- Hou et al. tested baricitinib efficacy in 10 SSc patients (6 female and 3 men with ILD detected at HRCT). The mean age was 41 years. All patients (9/9) were anti-Scl70 positive and 2/9 did not receive a previous immunosuppressive treatment while the others (7/9) were previously treated with CS (7/7), cyclosporine A (CsA, 2/7) and CYC (1/7) [23].

### 3.2. Motivation for JAKinibs Treatment Prescription and Response to Therapy

None of the papers analyzed introduced JAKinibs to control lung involvement. The main indications were uncontrolled skin involvement or musculoskeletal manifestations (Table 1).

- Deverapalli et al. reported the use of Tofacitinib to treat a patient with SSc with rapidly progressive skin involvement that caused functional impairment with reduced shoulders and hands range of motion and was complicated by digital ulceration. After just two weeks from the beginning of treatment with Tofacitinib, there was an improvement in the range of motion of the shoulder joints, a decrease in tightening of the skin over the dorsal hands and an initial healing of DUs. Over the course of a couple of months, re-pigmentation of some salt-and-pepper lesions of areas of the face was described [24];
- Fujita et al. analyzed the potential effectiveness of Baricitinib on articular involvement of a patient with SSc overlap Rheumatoid Arthritis that was refractory to multiple disease-modifying antirheumatic drugs. DAS28-CRP, Clinical Disease Activity Index (CDAI), and mRSS among others were recorded at baseline, 4, 24 and 52 weeks. At baseline the patient had an active articular disease with DAS28-CRP 7.61 and CDAI 57.2, skin involvement was mild with a mRSS 8/51. After one month, musculoskeletal manifestation showed a striking improvement with a DAS28-CRP of 3.00 and CDAI of 4. This improvement was well maintained at subsequent follow ups. Skin involvement also showed an improvement under Baricitinib treatment with a mRSS of 2 at 24 and 52 weeks [25];
- You H et al. describe the efficacy of Tofacitinib use in 10 patients with skin involvement refractory to conventional immunosuppressants. Patients had a modified Rodnan skin score (mRSS) that ranged from 11 to 33. Response to treatment was defined “clinically relevant” when a decrease in mRSS of more than 5 points or of more than 25% from baseline was evaluated. After a follow-up period of 3 and 6 months, response rate was 60% (6/10) and 83.3% (5/6), respectively. In general, eight patients met the response criteria, one showed an improvement in mRSS values without meeting response criteria, and one worsened at 6 months [21];
- Boleto et al. described the case of a patient with SSc overlap Rheumatoid Arthritis that was treated with Baricitinib for an active cutaneous and musculoskeletal disease, refractory to multiple conventional immunosuppressants. The patient was followed up regularly for 18 months. After two months from introduction of Baricitinib, the articular component showed a quick improvement in disease activity ccore 28 (DAS28-CRP) that was 4 points at baseline and 2.81 at two months. Articular effectiveness was maintained at subsequent follow ups. No effect was seen on mRSS that was 30/51 at baseline and 28/51 after 18 months [26];
- Karalilova et al. analyzed skin and musculoskeletal response to Tofacitinib treatment in 33 patients with SSc comparing them with a group of 33 patients with SSc cutaneous and articular manifestations that was instead treated with MTX. Together with mRSS variation, response was analyzed through grey scale, power doppler (in 10 joints), and skin thickness (in 5 anatomical sites) at ultrasound. After a follow-up period of 52 weeks, Tofacitinib showed a greater efficacy profile with respect to MTX with a mean percent of improvement of mRSS that was 44% higher than MTX. Moreover, Tofacitinib mean US skin thickness was statistically significantly lower than MTX. Tofacitinib showed a statistically significant better response also on the articular manifestations. The two drugs had a comparable safety profile [22].
- Kyriakou et al. studied Tofacitinib efficacy on skin and musculoskeletal involvement in a patient with SSc and axial Spondylarthritis (axSpA). Interestingly, the patient presented both axial involvement from AxSpA and peripheral arthritis from SSc. mRSS and Bath Ankylosing Spondylitis Disease Index Activity (BASDAI) score were recorded at baseline and after 6 months. Skin involvement showed a slight improvement with a mRSS that was 26 at baseline and 22 at follow up. BASDAI also improved being 7.4 at baseline and 4.8 at follow up. Moreover, an MRI of the sacroiliac joints was performed after 3 months of treatment revealing significant reduction of bone marrow oedema. No data were described regarding the peripheral articular manifestations, but the authors report that the patient reported a remarkable improvement also on that component [27];
- Hou et al. studied the effectiveness of Baricitinib treatment on skin involvement in a cohort of 10 patients with SSc. The occurrence of digital ulcers (DU) and mRSS were estimated at baseline, at week 12, and at week 24. Patients were classified as improvers in cases of a decrease in mRSS of more than 5 points and of more than 25% from baseline. One patient left the trial due to being incapable of attending follow-up visits at week 24. At baseline, 4 (40.0%) out of 10 patients had DU. At both follow ups, no new DU were reported. The mRSS of all nine patients that were evaluated at follow up improved more than 5 points, and 7 (77.78%) cases presented a decrease of more than 25% at week 24 [23].

### 3.3. ILD Response to JAKinibs

Since ILD response was not part of either the primary or the secondary outcomes of these studies, much data about this point were missing. PFTs were analyzed just by You et al., who reported no statistically significant change of FVC at 1, 3, and 6 months in the SSc-ILD subgroup treated with tofacitinib. Data about the diffusing capacity for carbon monoxide (DLCO) were not available. In this cohort, a HRCT scan was also performed at 3 or 6 months without showing either improvement or worsening [21]. Deverapalli et al. also considered PFTs at baseline, but no follow up was performed; the patient, however, remained stable at 2 months follow up [24].

HRCT scan was also available in several other studies:

- Karalilova et al. described that among the cohort of 13 patients with SSc-ILD treated with tofacitinib, only 1 patient withdrew from the study due to progressive ILD while the remaining 12 were stable at a 52-week follow up [22];
- Hou et al. performed a follow-up HRCT scan at baseline and at 24 weeks on 8/9 SSc-ILD patients and reported that 2 were significantly improved, 1 was slightly aggravated, and the other 5 remained stable. Respiratory symptoms were also analyzed through St. George’s Respiratory Questionnaire (SGRQ) at baseline and at 24 weeks in 8 patients. The authors recorded a significant improvement of respiratory symptoms after six months of treatment with Baricitinib with a statistically significant decrease in SGRQ score (*p* < 0.0079) [23];
- All the patients in the three case reports remained stable at follow up. Follow up duration varied according to the study (from 2 months to 52 weeks, as reported in Table 2).

### 3.4. Safety of JAKinibs Treatment in SSc Patients

As said, the studies were not dedicated just on the SSc-ILD population; for this reason, data regarding the safety of JAKinibs treatment in this particular population were not available. In the studies analyzed, one serious adverse event was described by Karalinova et al., who reported the development of progressive ILD in one patient that had to withdraw from the study. No other patient had to interrupt treatment due to side effects. Mild side effects were more common. You et al. [21] reported a single case of upper respiratory tract infection 3 weeks after using tofacitinib. Karalilova et al. [22] also described 6 cases of gastrointestinal intolerance (nausea, diarrhea), 4 cases of infections (1 urinary tract infection and 3 cases of viral infections), 4 cases of increased levels of transaminases (>1.5 x–<2.5 x), and 2 cases of increased level of cholesterol (>1.5 x). Hou et al. [23] reported a single case of herpes zoster infection. No side effects were reported in the case reports. No cardiovascular event was described in any of the studies analyzed.

## 4. Discussion

The analysis of these data shows that therapy with JAKinibs may lead to stabilization or improvement of SSc-ILD in the majority of cases. Worsening of ILD was seen in just 2 of the 31 cases found in the literature (6.5%).

The role of JAKinibs on ILD pathogenesis may be intimately linked to the JAK/STAT signaling pathway, which is crucial for several cellular processes and, when altered, may play a role in the development of inflammatory and autoimmune diseases. The JAK/STAT pathway is usually activated when a ligand, typically a cytokine (e.g., IL-4, IL-13, IL-6, IL-11, IL-31), growth factor (e.g., TGF-β1, EGF, PDGF, VEGF), or interferon, binds its receptor leading to its dimerization. Subsequently, receptor-associated JAKs get activated causing the phosphorylation of the tyrosine residues of the intracellular tail of their receptors, creating the docking sites for STATs. The activated STATs then translocate to the nucleus and modulate gene expression. Other non-canonical alternatives of this signaling pathway are also described in the literature [29].

**Figure 1:**
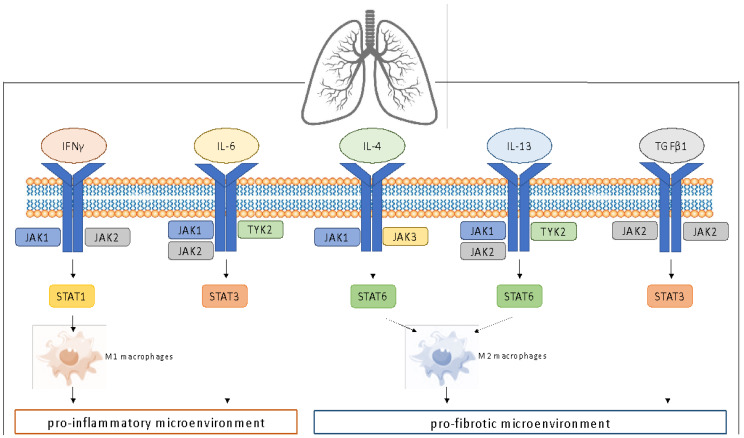
Schematic depiction of the main JAK/STAT pathways involved in developing pro-inflammatory and pro-fibrotic microenvironments.

Overexpression of pro-fibrotic and pro-inflammatory cytokines and growth factors is common in different ILDs [30]. Under this stimulation, the JAK/STAT pathway is triggered and promotes the polarization of the macrophages which, depending on their surrounding microenvironment and cytokine stimuli, can adopt various activation profiles (Figure 1).

Classical M1 macrophages have a pro-inflammatory phenotype. Their activation is the result of TLR4 and/or IFNγ signaling, strictly involving the JAK1/JAK2/P-STAT1 pathway through the activation of the IFNγ receptor. They are characterized by high secretion of pro-inflammatory cytokines and driving factors such as IL-6, CXCL10 and TNFα. This amplified secretion of IL-6 can also induce STAT3-mediated fibroblast-to-mesenchymal transition (FMT) [31], resulting in de-differentiation of fibroblasts into myofibroblasts with a mesenchymal phenotype, contributing to the development of ILD [32].

Alternative M2 macrophages instead have pro-fibrotic features and are activated by the IL-4 and/or IL-13 signaling that involves IL-4 receptor-JAK1/JAK3/P-STAT6 and IL-13 receptor-JAK1/JAK2/Tyk2/P-STAT6 associated pathways, respectively [20].

For these reasons JAK/STAT signaling represents a key process of macrophage polarization and JAKinibs, by targeting and impeding specific pro-inflammatory and pro-fibrotic pathways, could simultaneously exert anti-inflammatory and anti-fibrotic properties. This is the main reason for believing that they can represent a valid therapeutic option for SSc-ILD where these two pathogenetic processes—inflammation and fibrosis—coexist.

The studies that analyze JAK and STAT protein families’ expression amongst the different ILDs focus mainly on IPF. Through the analysis of animal models and human lung tissue, the most represented are JAK1, JAK2, and STAT1, STAT3 [33,34,35,36,37,38,39,40,41,42]. Between different JAK/STAT isoforms, JAK2/STAT3 appears to be predominant for the cellular changes observed in ILDs [30] and it is involved also in other diseases with a fibrotic phenotype (such as myelofibrosis, liver, myocardial, kidney, and skin fibrosis). JAK2/STAT3 pathway can be activated by several pro-fibrotic and pro-inflammatory factors such as TGFβ1, PDGF, vascular endothelial growth factor (VEGF), IL-6, IL-13, angiotensin II (AT2), 5-hydroxytryptamine (5-HT), and endothelin 1 (ET-1), all of them activated in IPF and Pulmonary hypertension (PAH) and involved in the development of pulmonary vasoconstriction (the case of 5-HT, ET-1, and AT2) [34].

To this point, studies evaluating the role of JAKinibs and JAK/STAT pathways in autoimmune diseases mainly investigated their anti-inflammatory effects, which are partly the results of their ability to modulate the activation state of pro-inflammatory macrophages by down-regulating IFNβ signature and IL-6 expression. Interestingly, Wang et al. [43] analyzed the activation of the fibrosis-associated IL6/JAK/STAT3 axis in skin and lung biopsies from SSc patients and the effects of Tofacitinib on skin and lung fibrosis of mice with bleomycin-induced skin and lung fibrosis. An aberrant activation of IL6/JAK/STAT3 and tofacitinib gene signatures was found in biopsies from SSc patients and Tofacitinib was reported to be effective in preventing bleomycin-induced skin and lung fibrosis in the animal models.

Clinical data regarding the efficacy of JAKinibs in SSc-ILD are sparse and seem associated with the stability of the pulmonary function and no ILD progression in 93.5% of patients [44]. Further data involving also larger cohorts are necessary to confirm these findings.

Interestingly, more data are available on JAKinibs efficacy in RA-associated ILD and they confirm what we detected in SSc [45]. A recent retrospective study [46] evaluated 75 RA patients comparing JAKinibs efficacy with ABA: 31 patients received treatment with a JAKinibs (18 were treated with baricitinib, 13 with tofacitinib) and RA-ILD progression was seen in 5 of them (16.1%), stability in 20 (64.5%), and improvement in 6 (19.4%). The group treated with ABA showed similar percentages (11.3%, 72.7%, and 16%, respectively) and differences were not statistically significant. Furthermore, a multicenter observational study [47] analyzed tofacitinib response in a cohort of 47 patients with RA-ILD and 387 patients with only RA (without ILD). Retention rates between the two groups were similar. After a median of 12 months follow up, PFTs in the RA-ILD group were stable in most patients.

Furthermore, the starting time of JAKinibs treatment could play a relevant role in the SSc-ILD response. In the studies analyzed, JAKinibs were often used in patients that were refractory to multiple treatments; however, as the promising role of JAKinibs treatment is the dual action on both the inflammatory and fibrotic components of the disease, an early start of the treatment could allow blocking the fibrotic changes before they lead to irreversible lung damage.

As scientific research on this field is progressing, it may be important to understand the optimal timing for the use of JAKinibs in SSc-ILD (also in combination with other drugs) and to determine whether the different specificity of each JAKinib could influence SSc-ILD response. Recent literature suggests that JAKinibs could also play a beneficial role in PAH, which is a severe complication increasing mortality [48]. JAK2 was overexpressed in pulmonary arteries of PAH-IPF patients in comparison to those with IPF and PAH. Furthermore, two experimental mice models investigated the efficacy of ruxolitinib on PAH (induced in the animal model with chronic hypoxia), showing an improvement in pulmonary arterial pressure levels, a partial reduction of right ventricular hypertrophy, and an almost complete restoration of the cardiac index without negative effects on cardiac function [49].

Finally, as described above, JAKinibs also present promising data on their effectiveness over other manifestations of the disease, such as on cutaneous and musculoskeletal involvement. As SSc is a systemic disease involving many different organs; the possible pluripotency of these drugs could be extremely helpful in allowing a broader control of the different manifestations with just one treatment. JAKinibs were in fact shown to be effective also on skin fibrosis and are being studied as a potential treatment of other fibrosing skin diseases as morphea [50]. In some of the studies analyzed, JAKinibs were also suggested to have a potential role on the control of digital ulceration, implying a hypothetical influence also on the vasculopathic component of the disease. All these effects need to be further studied on bigger cohorts with a more systematic approach to be confirmed but they allow us to understand why JAKinibs are considered to be an extremely promising treatment for SSc at the moment. JAKinibs also seem to present a promisingly good safety profile; however, more prospective studies are needed also to better investigate the possible higher risks of major adverse cardiovascular events and cancers that were found in a recent study on a cohort of patients with RA [51].

The present review’s main limitation is that the JAKinibs effect on ILD was neither a primary nor a secondary outcome in any of the studies. For this reason, many data were missing and consequently the strength of the results is low. Moreover, every study presented a different follow-up time, and this did not allow us to perform a precise comparison between them. Moreover, ILD response was not the main focus of the studies and the subanalysis of follow-up time, the safety of JAKinibs, and disease onset and duration were not possible. Therefore, the limited amount of data and of clinical trials significantly reduces the strength of the results. Publication bias is also another possible limitation of the study. Finally, another weakness is in the nature of the method: being a non-systematic review, it lacks a systemic approach and is, as a result, too subjective.

## 5. Conclusions

JAKinibs may represent a promising treatment in SSc-ILD as they target both the inflammatory and the fibrotic pathways that are fundamental for ILD development and progression. Possibly, early treatment with JAKinibs before fibrosis develops could yield better results; however, data on larger cohorts and randomized controlled trials (RCTs) are necessary to confirm these preliminary results.

## Figures and Tables

**Table 1:** Summary of the analyzed works.

Authors	Year of Publication	Patients n°	Mean Age	Treatment Duration	ILD (%)	Gender n° (%)	JAKinib Used	Dosage	Motivation for JAKinib Prescription
Deverapalli S. et al. [24]	2018	1	27	2 months	1 (100%)	M	TOF	5 mg bid	Skin and vascular involvement
Fujita Y. et al. [25]	2019	1	71	52 weeks	1 (100%)	F	BAR	4 mg die	Skin and musculoskeletal involvement
You H. et al. [21]	2020	10	51	6 months	6 (60)	F 8 (80)	TOF	5 mg bid	Refractory skin involvement
Boleto G. et al. [26]	2021	1	48	18 months	1 (100%)	M	BAR	4 mg die	Skin and musculoskeletal involvement
Karalilova R. et al. [22]	2021	33	49	52 weeks	13 (39%)	F 30 (91)	TOF	5 mg bid	Skin and musculoskeletal involvement
Kyriakou A. et al. [27]	2021	1	58	52 weeks	1 (100%)	F	TOF	N/A	Skin and musculoskeletal involvement
Hou Z. et al. [23]	2022	10	41	24 weeks	9 (90%)	F 6 (60)	BAR	4 mg die (2 pts), 2 mg die (7 pts)	Skin and vascular involvement

TOF: tofacitinib; BAR: baricitinib, bid: twice daily, pts: patients, N/A: not available.

**Table 2:** Pulmonary outcomes in the analyzed studies.

Authors	Stability/Total Patients *	TC Modification	FVC Stability
Deverapalli S. et al. [24]	1/1	N.R.	N.R.
Fujita Y. et al. [25]	1/1	1/1 stable	N.R.
You H. et al. [21]	6/6	6/6 stable	0
Boleto G. et al. [26]	1/1	N.R.	N.R.
Karalilova R. et al. [22]	12/13	1/13 worsened 12/13 stable	N.R.
Kyriakou A. et al. [27]	1/1	N.R.	N.R.
Hou Z. et al. [23]	8/9	1/8 worsened 2/8 improved 5/8 stable	N.R.

N.R.: Not Reported. * with “Stability” we identified either clinical and/or functional and/or radiological stability depending on the data available.
